# Common Sole Larvae Survive High Levels of Pile-Driving Sound in Controlled Exposure Experiments

**DOI:** 10.1371/journal.pone.0033052

**Published:** 2012-03-14

**Authors:** Loes J. Bolle, Christ A. F. de Jong, Stijn M. Bierman, Pieter J. G. van Beek, Olvin A. van Keeken, Peter W. Wessels, Cindy J. G. van Damme, Hendrik V. Winter, Dick de Haan, René P. A. Dekeling

**Affiliations:** 1 IMARES, IJmuiden, The Netherlands; 2 TNO, Den Haag, The Netherlands; 3 Defence Materiel Organisation, Den Haag, The Netherlands; 4 Ministry of Infrastructure and the Environment - DG Water, Den Haag, The Netherlands; Institute of Marine Research, Norway

## Abstract

In view of the rapid extension of offshore wind farms, there is an urgent need to improve our knowledge on possible adverse effects of underwater sound generated by pile-driving. Mortality and injuries have been observed in fish exposed to loud impulse sounds, but knowledge on the sound levels at which (sub-)lethal effects occur is limited for juvenile and adult fish, and virtually non-existent for fish eggs and larvae. A device was developed in which fish larvae can be exposed to underwater sound. It consists of a rigid-walled cylindrical chamber driven by an electro-dynamical sound projector. Samples of up to 100 larvae can be exposed simultaneously to a homogeneously distributed sound pressure and particle velocity field. Recorded pile-driving sounds could be reproduced accurately in the frequency range between 50 and 1000 Hz, at zero to peak pressure levels up to 210 dB re 1µPa^2^ (zero to peak pressures up to 32 kPa) and single pulse sound exposure levels up to 186 dB re 1µPa^2^s. The device was used to examine lethal effects of sound exposure in common sole (*Solea solea*) larvae. Different developmental stages were exposed to various levels and durations of pile-driving sound. The highest cumulative sound exposure level applied was 206 dB re 1µPa^2^s, which corresponds to 100 strikes at a distance of 100 m from a typical North Sea pile-driving site. The results showed no statistically significant differences in mortality between exposure and control groups at sound exposure levels which were well above the US interim criteria for non-auditory tissue damage in fish. Although our findings cannot be extrapolated to fish larvae in general, as interspecific differences in vulnerability to sound exposure may occur, they do indicate that previous assumptions and criteria may need to be revised.

## Introduction

The potential harmful impact of anthropogenic underwater sound on marine life is a growing concern. While most interest has focused on marine mammals, there is an increasing awareness of the possible effects on fish [1]–[4]. Loud impulse sounds, such as pile-driving sounds or seismic airgun blasts, may cause mortality by rupturing the swim bladder or other body parts [2], [5]–[6]. Exposure to anthropogenic sound may also cause permanent or temporary hearing loss [7]–[9], or physiological stress as indicated by increased cortisol levels [9]–[10] or increased heart rates [11]. Furthermore, anthropogenic sound may affect fish behaviour and distribution: avoidance (e.g. [12]), interference with intraspecific communication (e.g. [13]) and alterations of behavioural responses to acoustic signals (e.g. [14]) have been observed.

In view of the rapid extension of offshore wind farms, there is an urgent need to acquire more knowledge on the ecological benefits and adverse effects of offshore wind farm construction and operation [15]. Continuous sounds associated with operational wind farms and, in particular, loud impulse sounds associated with pile-driving for the construction of wind farms may have adverse effects on marine mammals and fish. Concern about the effects of pile-driving sound on fish has led to the formulation of interim criteria for non-auditory tissue damage by the US Fisheries Hydro-acoustic Working Group [16]. The agreed interim criteria define maximum peak sound pressure level at 206 dB re 1 µPa^2^ for all size of fish, maximum cumulative sound exposure level at 187 dB re 1 µPa^2^s for fish≥2 gram, and maximum cumulative sound exposure level at 183 dB re 1 µPa^2^s for fish < 2 gram. However, knowledge on the sound levels at which mortality or injury will occur is limited for juvenile and adult fish, and virtually non-existent for fish eggs and larvae [2]. While juvenile and adult fish may actively swim away from a sound source, planktonic larvae are passively transported by currents and are therefore not capable of avoiding sound exposure. As a result, fish larvae may suffer more from underwater sound than older life stages.

For an impact assessment of Dutch offshore wind farms, the effect of pile-driving sound on the number of larvae that reach the inshore nursery areas was modelled for 3 fish species [17]. An existing egg and larval transport model [18]–[20] was expanded with the assumption that egg and larval mortality might occur in a 1 km radius around a pile-driving site. This assumption was based on the limited information available at that time [17]. The results indicated that offshore pile-driving could cause a significant reduction in the number of fish larvae that reach the inshore nursery areas. The validity of this conclusion depends entirely on the validity of the underlying assumption, yet little is known about the vulnerability of fish eggs and larvae to pile-driving sound and the spatial scale at which mortality or injury may occur [2].

This study examined the effect of pile-driving sound on the survival of common sole (*Solea solea*) larvae. The first goal was to develop a laboratory set-up in which impulse sounds representative of pile-driving sound could be generated. The second goal was to use this laboratory set-up to determine the sound levels at which mortality in fish larvae might occur. The final series of experiments was preceded by a pilot series, in which the relevant exposure levels were explored and the required number of replicates per treatment was determined.

## Materials and Methods

### Larvaebrator

Exposure of fish larvae to pile-driving sound *in situ* is costly and logistically complicated, while reproduction of low frequency sounds in fish tanks or small basins is hampered by distortion due to reverberation and resonances [21]. Therefore, we decided to build a device specifically designed to enable controlled exposure of fish larvae to sound in a laboratory setting. This so-called ‘larvaebrator’ was inspired by an existing laboratory set-up for larger fish called the ‘fishabrator’ or the HICI-FT [22]–[23].

**Figure 1:**
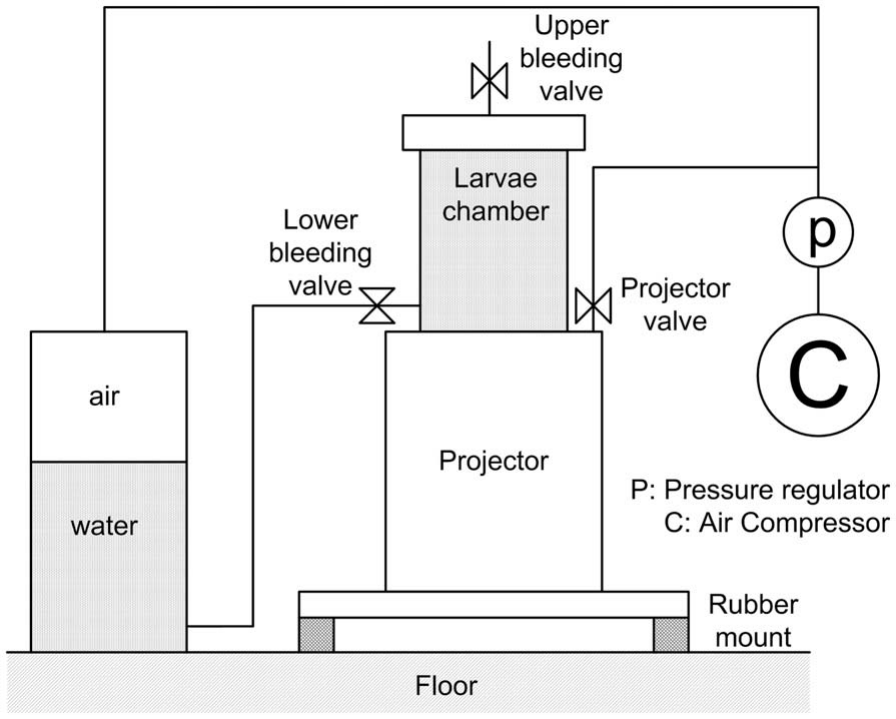
The ‘larvaebrator’ design. The larvaebrator is a device specifically designed to enable controlled exposure of fish larvae to sound in a laboratory setting.

The larvaebrator consists of an underwater sound source (LFPX-4 projector) on which a rigid-walled (28 mm thick steel), cylindrical chamber (110 mm diameter, 160 mm high) is placed (Figure 1). The chamber is filled with sea water (±1.25 litre) and up to 100 fish larvae can be placed in the chamber. The piston of the projector is also the bottom of the chamber and can directly excite the water with a given acoustic signal. Two configurations can be used; the test chamber is either completely filled with water, so that the projector mainly compresses the enclosed volume of water (pressure excitation), or a small layer of air is left at the top of the test chamber, so that the water in the chamber can move while compressing the air volume (velocity excitation). The chamber dimensions are much smaller than the shortest acoustic wavelength of interest (about 1.5 m at the maximum frequency of 1 kHz). Consequently, the larvae in the test chamber are simultaneously exposed to a homogeneously distributed sound pressure and particle velocity field. Sound pressure in the chamber is measured by four pressure transducers, mounted flush in the wall of the chamber. Sound particle velocity is measured by an accelerometer, mounted on the piston of the projector. A static pressure source (an air compressor) is incorporated in the device to enable applying static overpressure inside the chamber (Figure 1). The static overpressure can be varied between 0 and 3 bar, thus simulating a depth range of 0 to 30 m. The experiments in this study were carried out without static overpressure, because the greatest effect of sound pressure is expected to occur at a low static pressure (T. Carlson, unpublished results).

### Pile-driving sound

As it is unclear which characteristics of pile-driving sound could cause mortality, the acoustic signals to which the fish larvae were exposed had to be representative for actual sound exposures in the field. The actual exposure will vary with the properties of the pile-driving project and its environment. ‘Representativeness’ was achieved by playback of recorded pile-driving sound signals. Based on the initially assumed mortality range of 1 km [17], the playback level was adapted to the acoustic levels that were observed at distances between 100 m and 2 km from previous offshore wind farm construction projects in the Dutch part of the North Sea [24]–[25].

The playback level is defined in terms of acoustic metrics that quantify the received signals [26]. Studies on the impact of underwater sound on marine life [16], [23], [27] quantify impulsive sound in terms of sound exposure level (in dB re 1 µPa^2^s per strike and/or cumulative) and zero to peak sound pressure (value in Pa or level in dB re 1 µPa^2^). Other possible metrics (impulse, rise time, peak to peak sound pressure, kurtosis, etc.) have sometimes been suggested, but the associated dose-response relations are even less clear than for sound exposure level and zero to peak pressure [2]. Therefore the sound pressure metrics used in the present study were zero to peak sound pressure and sound exposure level. Similar metrics can be derived for acoustic particle velocity. Although sound particle velocity has a direction associated to it, the metrics proposed here only concern the magnitude of particle velocity.

The sound metrics were defined as follows:

• *Zero to peak sound pressure* is the maximum absolute value of the unweighted instantaneous sound pressure in the measurement bandwidth. *Zero to peak sound pressure level* (L_z−p_) is ten times the logarithm to the base 10 of the ratio of the square of the zero to peak sound pressure to the square of the reference sound pressure of 1 µPa.
• *Sound exposure* is the time integral of the time-varying square of the unweighted instantaneous sound pressure in the measurement bandwidth over the duration of a single piling impact. *Single strike sound exposure level* (SEL_ss_) is ten times the logarithm to the base 10 of the ratio of the sound exposure of a single piling impact signal to the reference sound exposure of 1µPa^2^s. *Cumulative sound exposure level* (SEL_cum_) is the summation over a specified number of piling impacts; SEL_cum_ is the average SEL_ss_ plus ten times the logarithm to the base 10 of the number of strikes.
• *Zero to peak sound particle velocity* is the maximum absolute value of the unweighted instantaneous total sound particle velocity in the measurement bandwidth. *Zero to peak sound particle velocity level* (L_v,z−p_) is ten times the logarithm to the base 10 of the ratio of the square of the peak sound particle velocity to the square of the reference sound particle velocity of 1 nm/s.
• *Sound particle velocity exposure* is the time integral of the time-varying square of the unweighted instantaneous sound particle velocity in the measurement bandwidth over the duration of a single piling impact. *Single strike sound particle velocity exposure level* (VEL_ss_) is ten times the logarithm to the base 10 of the ratio of the sound exposure to the reference sound particle velocity exposure of 1 (nm/s)^2^s. *Cumulative sound particle velocity exposure level* (VEL_cum_) is the summation over a specified number of piling impacts; VEL_cum_ is the average VEL_ss_ plus ten times the logarithm to the base 10 of the number of strikes.

The sound measured at 100 m from pile-driving events in the North Sea (OWEZ wind farm, 4 m diameter steel monopole, at a water depth of ±20 m, with hammer strike energy of ±800 kJ) had a broadband L_z−p_ up to 210 dB re 1 µPa^2^ and a broadband SEL_ss_ up to 188 dB re 1 µPa^2^s [25]. Propagation loss to various distances depends in a complex manner on water depth (bathymetry), condition of the water surface (waves) and the acoustic properties of water and sediment. For North Sea conditions in 20–25 m deep water with a sandy bottom, distances between 100 m and 2 km from the pile are approximately in the ‘mode-stripping’ region [28]. In this region, propagation loss for low frequency pile-driving sound approximately varies with distance R as 15log_10_R. Thus, the levels at 2 km distance are estimated to be about 20 dB lower than the levels at 100 m (i.e. SEL_ss_  =  168 dB re 1µPa^2^s and L_z−p_  =  190 dB re 1 µPa^2^ at 2 km).

At distances ≥ 100 m from the pile in 20–25 m deep water, the acoustic particle velocity level is roughly proportional to the acoustic pressure level through the characteristic impedance of the medium (ρc): particle velocity level equals pressure level minus 20log_10_(ρc•(10^6^•10^−9^)) ≈ 64 dB re 1 (nm/s/µPa)^2^. This approximation includes a correction factor that accounts for the different reference units for pressure and velocity. Hence, broadband L_v,z−p_ between 127 and 147 dB re 1 (nm/s)^2^ and broadband VEL_ss_ between 104 and 124 dB re 1 (nm/s)^2^s corresponds with the estimated values for L_z−p_ and SEL_ss_ at distances between 100 m and 2 km from the pile.

Two single strike signal recordings were selected for playback, one measured at 100 m and one measured at 800 m distance from the pile. The recorded signals were scaled to different levels to simulate different distances from the pile, the 100 m signal was used for distances between 100 and 800 m, the 800 m signal was used for distances ≥ 800 m.

Typical recorded SEL_ss_ spectra [25], [29] show that the main (unweighted) energy of underwater pile-driving sound is generated in the 50 Hz to 1 kHz bands. The playback sound was limited to this frequency band, to avoid excitation of spurious resonances in the larvaebrator.

**Figure 2:**
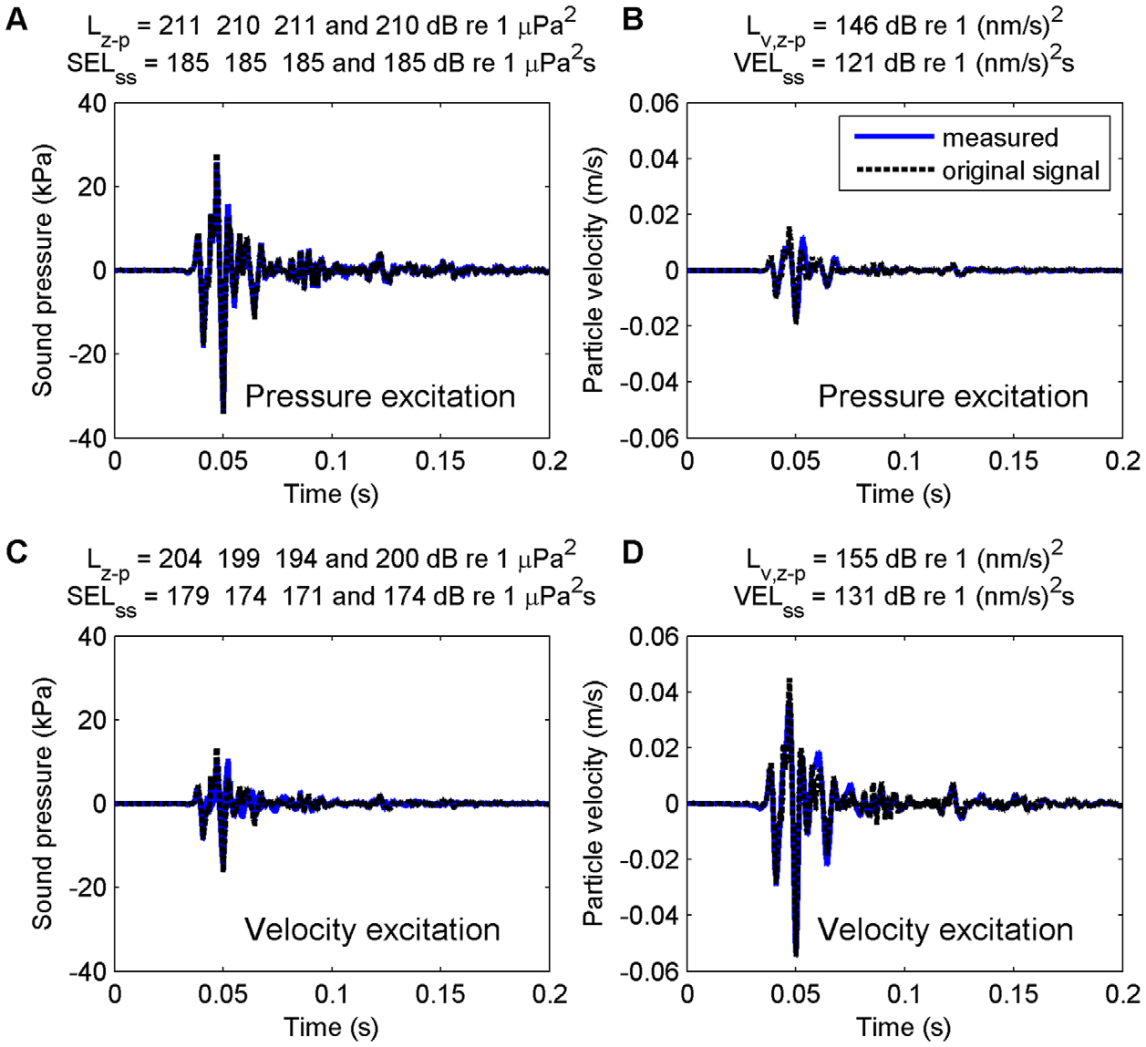
Comparison of the original and measured signal shape. Comparison of the original signal shape (recorded in the field) and the observed signal shape (measured in the larvaebrator) for a pressure excitation (A, B) and a velocity excitation (C, D), in terms of sound pressure (A, C) and sound particle velocity (B, D). The original signal is scaled to match the peak of the measured signal. The sound levels are given in the header of each panel.

Measurements showed that the projector reproduced the original recorded signal shape quite accurately for sound pressure in the pressure excitation configuration, and for particle velocity in both excitation configurations (Figure 2). The velocity levels were substantially higher for a velocity excitation compared to a pressure excitation. Hence, the effect of particle velocity could be examined decoupled from the effect of sound pressure. In case of pressure excitation, however, the velocity levels were higher than expected from compression of the water volume alone, probably due to remaining flexibility (air/membrane) in the chamber. This means that the set-up does not enable examination of the effect of sound pressure decoupled from particle velocity. The observed pressure to velocity ratio was actually close to the ratio in a plane wave in unbound water. In a plane wave, the acoustic particle velocity and acoustic pressure levels are approximately related through the characteristic impedance of the medium (see above). The measured L_z−p_ of 211 dB re 1µPa^2^ (Figure 2a) corresponded with an expected free field L_v,z−p_ of 147 dB re 1 (nm/s)^2^ and an observed L_v,z−p_ of 146 dB re 1 (nm/s)^2^ (Figure 2b). The measured SEL_ss_ of 185 dB re 1 µPa^2^s corresponded with an expected and observed VEL_ss_ of 121 re 1 (nm/s)^2^s. Hence, the pressure excitation exposures represent realistic pressure to velocity ratios.

**Figure 3:**
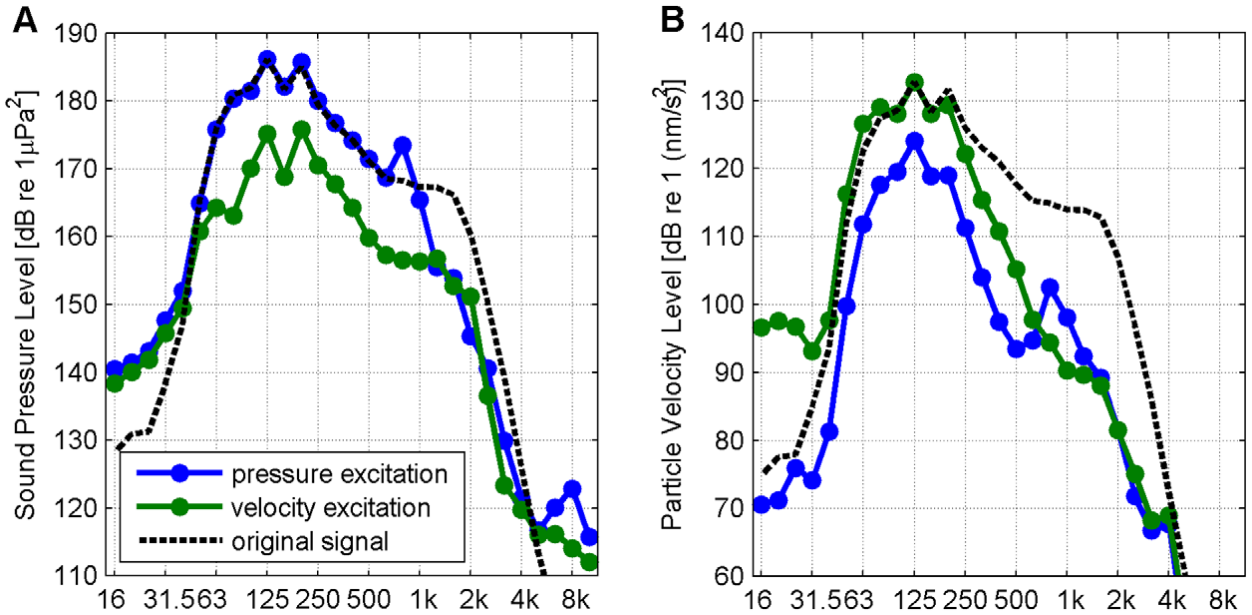
Comparison of the original and measured frequency spectra. Mean square sound pressure level spectrum (A) and particle velocity level spectrum (B) in 1/3-octave bands (averaged over 0.2 s intervals) for a pressure excitation, a velocity excitation and the original signal scaled to match the peak of the measured signal.

The main characteristics of the frequency spectra of pressure and velocity are reproduced to an acceptable level (Figure 3). The reproduced sound particle velocity spectrum at frequencies above 250 Hz is lower than the spectrum of the recorded sound, but the dominant energy in the range between 63 and 250 Hz is reproduced correctly.

### Larvae

Common sole (*Solea solea*) is a commercially important flatfish species, which was included in the impact assessment of Dutch offshore wind farms [17]. For most marine fish species, it is difficult to obtain eggs or larvae, but common sole eggs and larvae could be obtained throughout the year from a commercial hatchery (SOLEA). Fertilised eggs were purchased from the hatchery and reared to the required larval stage in large cultivation chambers in the laboratory. As the effect of sound exposure may vary between larval stages related to the development of organs, different larval stages were used in the experiments. Stage identification was based on the following classification [30]:

Stage 1: Yolk sac present

Stage 2: Yolk sac absorbed, development of spines and swim bladder.

Stage 3: Swim bladder fully inflated, appearance of fin rays, notochord straight

Stage 4: Onset of asymmetry and eye migration, notochord bent

 Stage 4a: Notochord caudally bent upwards by < 45°,  eyes symmetrical

 Stage 4b–d: Notochord bent by ≥ 45°, onset of eye  migration

Stage 5: Completion of metamorphosis, swim bladder resorbed.

**Figure 4:**
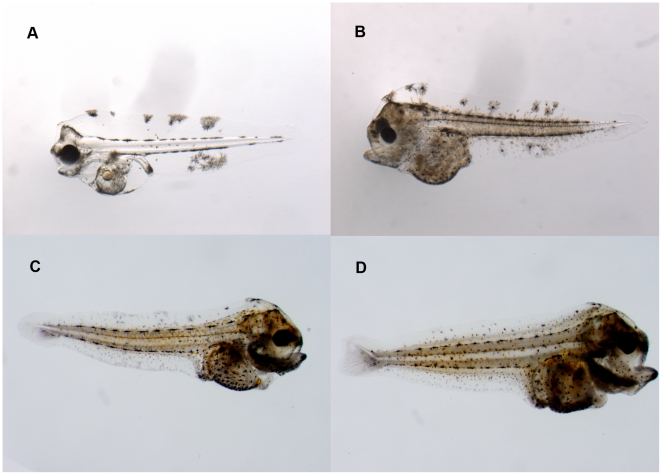
The larval stages of common sole (*Solea solea*) that were used in the experiments. The photos show a stage 1 larva of 5.3 mm (A), a stage 2 larva of 6.0 mm (B), a stage 3 larva of 6.5 mm (C) and a stage 4a larva of 7.1 mm (D).

Three (groups of) larval stages were used in the experiments: 1, 2 and 3–4a (Figure 4). The late larval stages were not included because by then the larvae disappear from the water column related to the transition from a pelagic to a demersal life style [31]–[32].

Development rates depend on temperature [33]–[35]. The water temperature in the cultivation chambers was slowly raised from the temperature in the hatchery (12°C) to the ambient temperature in the laboratory (16°C). Within this range, the temperature was manipulated so the majority of larvae would be in the required developmental stage on the days that the treatments were applied. Variations in development rates were observed between larvae that were reared at the same temperature; larvae originating from one spawning event and reared at the same temperature could range from stage 3 to stage 4a.

**Figure 5:**
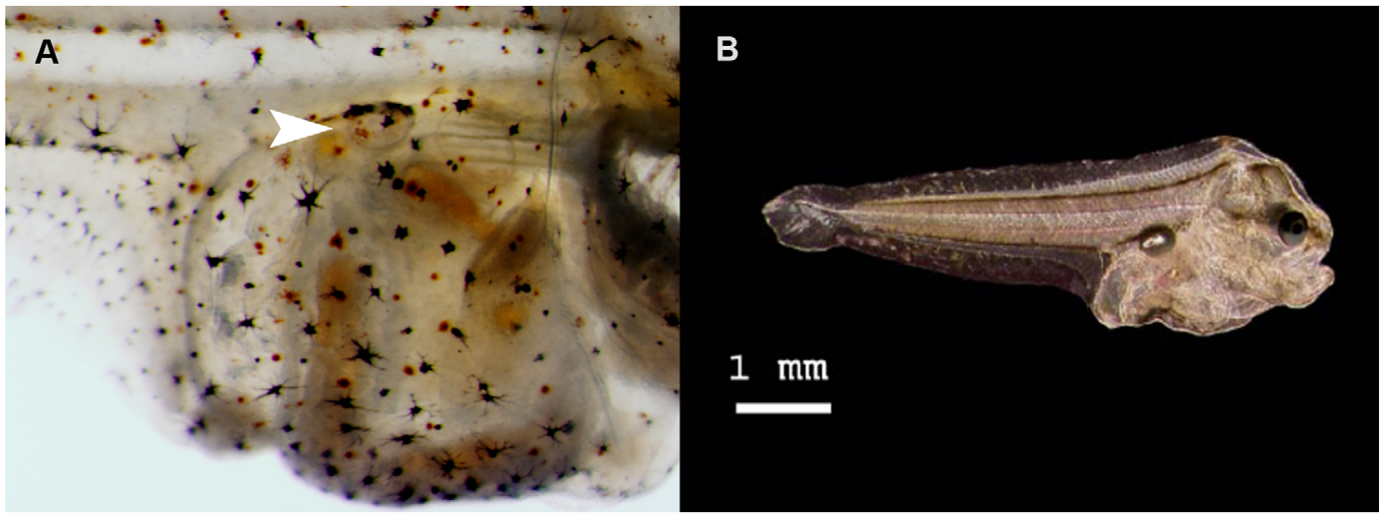
The swim bladder in common sole (*Solea solea*) larvae. The swim bladder in a stage 4a larva as observed in this study (A) and a published image [36] of the swim bladder in a stage 4a larva (B).

In stage 3–4a larvae, inflated swim bladders (Figure 5a) were observed in most, but not all larvae. Similar observations were done previously for common sole [36]–[37]. In an aquaculture study [36], inflated swim bladders were observed at 16 days after hatching in larvae reared at 18°C (Figure 5b), but not all larvae of that age had an inflated swim bladder. Histological examination of larvae reared at 19°C showed that the gas gland and bladder are already developed 5 days after hatching, the first inflated swim bladders appear at 10 days after hatching, and not all larvae have an inflated bladder during the inflation period [37]. They observed a dilated pneumatic duct when the swim bladder begins to inflate, indicating passage of gas from the digestive tract to the swim bladder (i.e. a physostomous swim bladder), but they also found indications that inflation may be realised by gas secretion of the gas gland.

### General procedures

Each experiment consisted of a treatment followed by a monitoring period. A treatment was either a sound exposure or a control. The water in the test chamber of the larvaebrator was refreshed before each treatment. Water temperature in the test chamber was the same as in the cultivation chambers. For each experiment, 25 (±5) larvae were taken from the cultivation chambers and subjected to treatment. After treatment, each batch of larvae was transferred to a separate ‘batch-container’ and held during the monitoring period. The control groups underwent the same handling procedures as the exposure groups. The larvae were transferred to and from different water bodies using a plastic pipette, from which the tip was cut off to enlarge the opening. This method minimises mortality due to handling, but it is time consuming as only one or two larvae can be transferred at the same time. The total duration of a treatment including handling of the larvae was 15 (±5) minutes.

From 3–4 days after hatching onwards (i.e. larval stage 2+), the larvae were fed daily and *ad libitum* with *Artemia*. The water in the batch-containers was refreshed every day. The response variable that was measured was mortality; the numbers of dead and live larvae in each batch were counted directly after the treatment and daily during the monitoring period. Dead larvae disintegrated completely within 24 hours. Recently dead larvae were visually recognized by their shape or immobility. Within a few hours after death, a larva shrivels up and its shape clearly indicates that it is dead. Immobile larvae were examined using a stereomicroscope to check heart-beat and respiratory activity. Dead larvae were removed from the batch-containers.

The batch-containers were coded and, except for the observations directly after the treatments, the person scoring mortality was not aware of the treatment belonging to the code. The treatments within each replication round were applied in random sequence to avoid bias due to potential serial effects.

This study was performed in accordance with Dutch law concerning animal welfare. The protocol was approved by the Animal Ethical Commission (DEC) of Wageningen UR (experiment code 2010085 under application 2010063.c).

### Pilot experiments

In a pilot series of experiments, we maximised the number of treatments and, consequently, minimised the number of replicates per treatment, because very little is known about critical values for sound exposure with regard to larval survival. Each of the three larval stages was subjected to several exposures (Tables 1 and 2) and a control treatment. Two replicates per treatment were carried out for stage 1 larvae, four replicates for stage 2 larvae and five replicates for stage 3–4a larvae. Mortality was recorded directly after the treatment and daily until 10 days after the treatment.

**Table 1:** Sound levels of the pressure excitation exposures applied in the pilot experiments.

Stage	Measured sound levels			Strikes	Distance (m)
	L_z−p_	SEL_ss_	SEL_cum_		
	(dB re µPa^2^)	(dB re1 µPa^2^s)	(dB re µPa^2^s)		
1	198	175	175	1	800
	211	187	187	1	100
	211	187	204	50	100
2	206	181	204	200	200
	210	186	203	50	100
	210	186	206	100	100
3–4a	205	181	206	300	200
	210	186	196	10	100
	210	186	206	100	100

L_z−p_  =  zero to peak sound pressure level, SEL_ss_  =  single strike sound exposure level and SEL_cum_  =  cumulative sound exposure level, see the text for further explanation. The last 2 columns present the corresponding distance from a ‘typical’ North Sea pile-driving installation and number of strikes.

**Table 2:** Sound levels of the velocity excitation exposures applied in the pilot experiments.

Stage	Measured sound levels			Strikes	Distance (m)
	L_v,z−p_	VEL_ss_	VEL_cum_		
	(dB re 1 (nm/s)^2^)	(dB re 1 (nm/s)^2^s)	(dB re 1 (nm/s)^2^s)		
1	133	111	111	1	800
	148	125	125	1	100
	147	124	144	100	100
2	142	118	141	200	200
	147	122	142	100	100
3–4a	145	122	147	300	200
	148	125	145	100	100

L_v,z−p_  =  zero to peak sound particle velocity level, VEL_ss_  =  single strike sound particle velocity exposure level and VEL_cum_  =  cumulative sound particle velocity exposure level, see the text for further explanation. The last 2 columns present the corresponding distance from a ‘typical’ North Sea pile-driving installation and number of strikes.

Two types of sound exposure were applied: pressure excitation or velocity excitation (see above). The larvae were exposed to single or multiple strikes at different levels of sound pressure or particle velocity (Tables 1 and 2). The maximum SEL_cum_ possible with the larvaebrator (using recorded pile-driving sounds) was 206 dB re 1 µPa^2^s, which corresponded to 100 strikes at a distance of 100 m from a ‘typical’ (as described above) North Sea pile-driving installation. The strike rate was 50 strikes per minute, so an exposure to 100 strikes lasted 2 minutes.

### Final experiments

In the final series of experiments, the number of replicates per treatment was substantially increased, to obtain a higher precision on the estimates of differences in mortality between treatments. The results of the pilot series were used in a power analysis to estimate the number of replicates required for sufficient power (i.e. probability of detecting an effect significantly at the 95% level, given a certain sample size and experimental design) to detect a ‘50% effect’. The % effect was defined as 100% (p_e_−p_c_)/(1−p_c_), in which p_e_ is the estimated mean probability of death in the exposure group and p_c_ is the estimated mean probability of death in the control group. Note that with this definition of the effect to be detected, the difference between the exposure group and control group depends on the mortality in the control group. The analysis showed that doubling the number of replicates increased the power far more than doubling the number of larvae per replicate. Fifteen replicates for each treatment, with 25 larvae per batch, were estimated to give a high probability (≥ 96%) of detecting a 50% effect significantly (at the 95% level) after 5 days.

**Table 3:** Sound levels of the pressure excitation exposures applied in the final experiments.

Stage	Measured sound levels			Strikes	Distance (m)
	L_z−p_	SEL_ss_	SEL_cum_		
	(dB re 1 µPa^2^)	(dB re 1 µPa^2^s)	(dB re 1 µPa^2^s)		
1	205	181	201	100	200
	210	186	206	100	100
2	205	180	200	100	200
	209	185	205	100	100
3–4a	205	181	201	100	200
	209	185	205	100	100

L_z−p_  =  zero to peak sound pressure level, SEL_ss_  =  single strike sound exposure level and SEL_cum_  =  cumulative sound exposure level, see the text for further explanation. The last 2 columns present the corresponding distance from a ‘typical’ North Sea pile-driving installation and number of strikes.

Given the resources available, it was possible to carry out 3 treatments (2 exposures and 1 control) with 15 replicates for each of the 3 larval stages. We decided to focus on pressure excitation exposures as these appeared to have an effect (although non-significant) in the pilot series. The same two exposures were used for all larval stages: the highest sound pressure exposure possible with the larvaebrator (using recorded pile-driving sounds) and an exposure which was approximately 5 dB lower in both SEL_cum_ and L_z−p_ (Table 3).

As both the absolute level of mortality in the control group and the variation in mortality between batches with the same treatment increased over time, the statistical power to detect a 50% effect decreased with the duration of monitoring. Therefore, the monitoring period was reduced to 7 days in the final series of experiments.

### Statistical analysis

Estimates of mortality per larval stage and treatment, as well as the statistical significance of differences between exposure and control groups, were calculated using a generalised linear mixed model. This model treats the data (death or survival of a larva) as outcomes of binomial trials in which the probability of death is a function of treatment, and takes account of possible random variation in mortality between batches (termed ‘batch effect’ hereafter). It is necessary to account for such batch effects because, if present, the assumption (under the binomial distribution) that the outcomes of larvae are determined independently of one another is violated.

The statistical model was formulated as follows:

• The logit transformed probabilities of death *p_i,j_* (in treatment *i* and batch *j*) were modelled as a function of treatment and random batch effect (*α_j_*):

logit(p_ij_)  =  treatment_i_+α_j_.

• The numbers of dead larvae in batch *j* from treatment *i* (*k_ij_*) were assumed to be binomially distributed depending on the probability of death (*p_ij_*) and the number of larvae at the beginning of the experiment (*N_ij_*, usually 25):

k_ij_ ∼ Bin(p_ij_, N_ij_).

• The random batch effects (*α_j_*) were assumed to be normally distributed with mean zero and variance σ^2^:

α_j_ ∼ N(0, σ^2^).

The model was fitted and statistical significance tests were performed using the glimmix procedure (with the Kenward-Roger approximation for the degrees of freedom) in SAS (SAS/STAT software. SAS Institute Inc., Cary, North Carolina, USA). The model was fitted separately to the data for each larval stage and for each of two monitoring periods (5 or 7 days in the final series, 5 or 10 days in the pilot series). If, for a given larval stage and monitoring period, the variance of the batch effect was estimated to be (near) zero, then the model was reduced to a generalised linear model without a batch effect.

## Results

In the pilot series, no immediate effect of sound exposure (directly after treatment) was observed for any of the three larval stages. Mean mortality in the control group increased from 0% directly after treatment to 67% at the end of the 10 day monitoring period for larvae that were in stage 1 at the time of the treatment. This was 0 to 59% for stage 2 larvae, and 0 to 10% for stage 3–4a larvae. In the case of stage 2 larvae, mortality in the control group was clearly lower than mortality in the highest sound pressure exposure group (SEL_cum_  =  206 dB re 1 µPa^2^s): mean mortality after 10 days was 78% in the exposure group compared to 59% in the control group, that is ±50% less survivors in the exposure group. This difference was not statistically significant, possibly due to low statistical power (i.e. too few replicates). No indications for an effect of sound exposure were observed in the other larval stages or at other sound levels. High variability in mortality between batches with the same treatment was observed.

**Figure 6:**
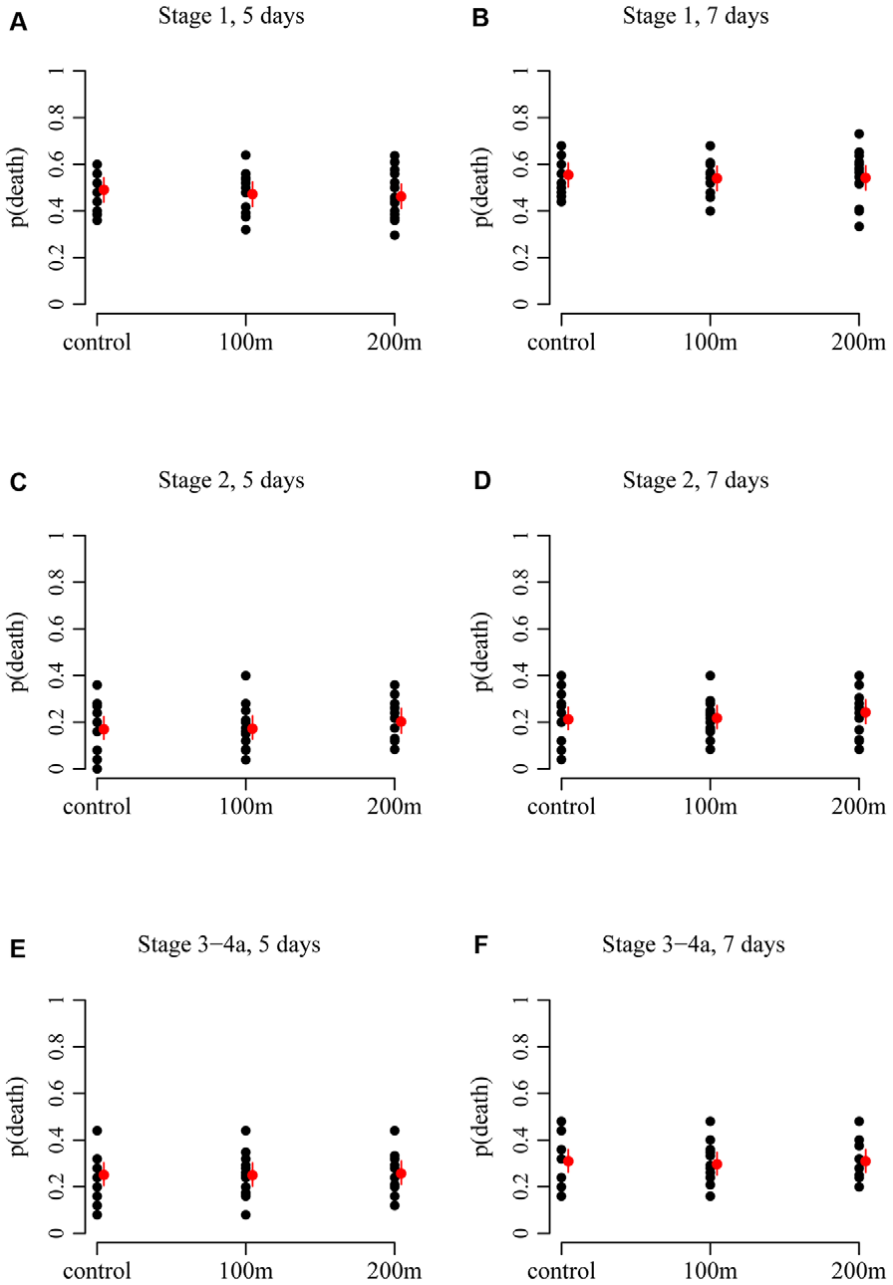
Mortality by larval stage and treatment at 5 and 7 days after the treatment. Estimated mean probability of death with 95% confidence limits (red symbols and bars) and observed mortality for each replicate within each treatment (black symbols). Each replicate consisted of 25 (±5) larvae. The labels of the sound exposure treatments refer to the distance from the pile, the associated sound levels are presented in Table 3.

In the final series, as in the pilot series, no immediate effect of sound exposure was observed for any of the three larval stages. Mean mortality in the control group increased from 0% directly after treatment to 55% at the end of the 7 day monitoring period for stage 1 larvae, from 0 to 21% for stage 2 larvae, and from 0 to 31% for stage 3–4a larvae. No clear differences between the exposure groups and the control group were observed for any of the larval stages (Figure 6). The factor treatment was statistically insignificant for all larval stages (Table 4).

**Table 4:** Analysis of variance of the probability of death modelled as a function of treatment and random batch effect.

Stage	Days	Chi^2^/DF	Random effect	Fixed effect			
			variance	Num DF	Den DF	F value	Pr > F
1	5	0.82	0	2	42	0.31	0.7
	7	0.68	0	2	42	0.09	0.9
2	5	1.00	0.1404	2	41.90	0.48	0.6
	7	1.00	0.0568	2	41.67	0.40	0.7
3–4a	5	0.99	0.0340	2	42	0.03	0.9
	7	0.99	0	2	42	0.10	0.9

Model estimates of the 95% confidence interval for the difference between exposure and control were used to estimate the effect that could have been detected with these experiments. Estimates of the upper limit of the 95% confidence interval for effect ranged from 8 to 14% (Table 5). This means that the probability of an effect larger than 14% was insignificant (< 5%). Hence, the detectable effect was substantially smaller than the 50% aimed for in the power analysis.

**Table 5:** Model estimates of the mean and 95% confidence limits for probability of death in each treatment and for the effect of exposure.

Stage	Days	Treatment	Estimated probability of death			Estimated effect	
			mean	lower limit	upper limit	mean	upper limit
1	5	100 m	0.47	0.42	0.52	−4%	11%
		200 m	0.46	0.41	0.51	−6%	9%
		control	0.49	0.44	0.54		
1	7	100 m	0.54	0.49	0.59	−3%	13%
		200 m	0.54	0.49	0.59	−3%	14%
		control	0.55	0.50	0.61		
2	5	100 m	0.17	0.13	0.23	0%	10%
		200 m	0.20	0.15	0.26	4%	14%
		control	0.17	0.13	0.22		
2	7	100 m	0.22	0.17	0.27	1%	10%
		200 m	0.24	0.19	0.30	4%	14%
		control	0.21	0.17	0.26		
3–4a	5	100 m	0.25	0.20	0.30	0%	10%
		200 m	0.26	0.21	0.31	1%	11%
		control	0.25	0.21	0.30		
3–4a	7	100 m	0.30	0.25	0.35	−2%	8%
		200 m	0.31	0.26	0.36	0%	10%
		control	0.31	0.26	0.36		

The effect of exposure was defined as 100% • (p_e_−p_c_)/(1−p_c_), in which p_e_ is the estimated mean probability of death in the exposure group and p_c_ is the estimated mean probability of death in the control group. The labels of the sound exposure treatments refer to the distance from the pile, the associated sound levels are presented in Table 3.

## Discussion

Experimental exposure of common sole larvae to pile-driving sound levels up to SEL_cum_  =  206 dB re 1 µPa^2^s and L_z−p_  =  210 dB re 1 µPa^2^ did not result in increased mortality during the first 7 days after exposure. No statistically significant differences in mean mortality were found between the control and exposure groups for any of the larval stages. Standard errors on mortality estimates were such that an exposure effect of more than 14% could be excluded at the 95% confidence level.

For larvae not exposed to sound (i.e. the control groups), mean cumulative mortality after 7 days ranged from 8 to 56%. These levels were not considered to be high compared to natural mortality. Natural larval mortality rates are usually expressed in instantaneous daily mortality rates (Z in the equation N_t_  =  N_0_ • e^−Zt^, N_0_ is number of larvae at t  =  0 days and N_t_ is number of larvae after t days). Published estimates for European flatfish species range between 0.035 d^−1^ [38] for sole in the Bristol Channel and 0.08 d^−1^ [39] for plaice in the North Sea, that is 22–43% mortality after 7 days. Similar or higher larval mortality rates were estimated for other marine fish species [40]. The differences in control group mortality were not only related to larval stage, but also to spawning stock quality. Clear differences were observed in the viability of eggs and larvae obtained from different spawning events. This was also reported for hatchery reared common sole larvae [36]; mortality ranged from 35 to 80% depending on the spawning group.

The interim SEL_cum_ criterion defined by the US Fisheries Hydro-acoustic Working Group for non-auditory tissue damage in small fish (< 2 g) is 183 dB re 1 µPa^2^s [16]. The highest SEL_cum_ used in the present study (206 dB re 1 µPa^2^s) was much higher than this norm, but no significant effects on the survival of common sole larvae were found. Actually, very little is known on the sound levels that cause damage or mortality in fish eggs and larvae. No studies have addressed the effect of pile-driving sound on fish larvae, and only a few studies have investigated the effect of low frequency, loud impulse sounds on fish larvae [2].

The effect of seismic air gun sounds on eggs and different larval stages of cod (*Gadus morhua*), saithe (*Pollachius virens*), herring (*Clupea harengus*), turbot (*Psetta maximus*) and plaice (*Pleuronectes platessa*) was examined in field experiments [41]. Effect was related to the distance from the sound source and the corresponding L_z−p_ ranged from 220 to 242 dB re 1 µPa^2^. Cod, turbot and herring larvae were examined in the yolk sac stage: cod showed a small but insignificant effect at 242 dB, herring showed no significant effects due to overall high mortality rates, and turbot showed significant effects at all levels of exposure. Cod and saithe were examined in the post yolk sac larval stages: significant effects were observed for cod at exposures ≥ 223 dB, no significant effects were observed for saithe due to overall high mortality rates. Cod, turbot, herring and plaice were examined in the post-larval stage: cod showed a significant effect at 242 dB, small but insignificant effects were observed at the higher sound levels for the other 3 species. The authors also observed damage to the neuromasts of the lateral line system and to other organs in cod and turbot larvae [41].

Larval and small juvenile spot (*Leiostomus xanthurus*) and pinfish (*Lagodon rhomboides*) were exposed to blast shock waves in field experiments [42]. The size of the test animals was 18–20 mm for spot and 16–17 mm for pinfish (note that these larvae/juveniles were larger than the larvae used in the present study). The authors recorded death, lethal and sub-lethal injuries within 24 hours after exposure. For spot, the proportion dead or injured was 0% in the control group and 100% at the highest exposure level: zero to peak pressure  =  278−692 kPa (L_z−p_ ≈ 229−236 dB re 1 µPa^2^) and energy flux density  =  1.096−3.642 J m^−2^ (SEL_ss_ ≈ 182−187 dB re 1 µPa^2^s assuming the impedance of the medium to be 1.53•10^6^ kg/m^2^s). For pinfish, the proportion dead or injured was 0% in the control group and ranged from 33–100% at the highest exposure level: zero to peak pressure  =  558−866 kPa (L_z−p_ ≈ 235−239 dB re 1 µPa^2^) and energy flux density  =  1.311−2.594 J m^−2^ (SEL_ss_ ≈ 183−186 dB re 1 µPa^2^s ). The blasts applied in this study apparently had a different signal shape compared to our playback of pile-driving sounds; their highest exposures had much higher zero to peak pressure levels then in our study, whereas the single-strike sound exposure levels were comparable.

These two studies show that exposure to loud impulse sounds can cause lethal and sub-lethal effects in fish larvae. The zero to peak pressure levels applied in these studies were 12 to 32 dB higher than in the present study. SEL_ss_ was only reported in one of the two studies [42] and their highest levels (182–187 dB re 1 µPa^2^s) were comparable to the levels we used in the final series of experiments (181–186 dB re 1 µPa^2^s). This indicates that either L_z−p_ may be a more critical metric for mortality than SEL_cum_, or that common sole larvae are less vulnerable to sound exposure than pinfish and spot larvae/small juveniles.

The swim bladder is an organ which is sensitive to sound pressure and it has been suggested that fish with swim bladders are more vulnerable to sound exposure than species that do not have such air chambers [2]. Common sole larvae only have a swim bladder during a limited period of their larval life [36]–[37]. This may be the reason for the absence of significant effects in stage 1 and 2 larvae. However, significant effects of sound (at higher levels than those used in the present study) have been observed in yolk sac turbot larvae [41], and these larvae do not have a swim bladder either [30]. If the presence of a swim bladder is critical at the sound exposure levels used in this study, then an effect could have been expected in stage 3–4a larvae. Visual inspection before treatment showed that most of these larvae had an inflated swim bladder, but we cannot exclude gas loss from the swim bladder due to handling prior to exposure.

Statistically significant lethal effects of exposure to pile-driving sounds in common sole larvae could occur at higher sound levels than the highest levels used in the present study (SEL_cum_  =  206 dB re 1 µPa^2^s, L_z−p_  =  210 dB re 1 µPa^2^). The limited information available to date indicates that interspecific differences in vulnerability to sound exposure may occur. Hence, we would not recommend that the conclusion based on common sole larvae be broadly extrapolated to other fish larvae. However, this study does indicate that the previous assumptions [17] and interim criteria [16] may need to be revised.

Studies on the effects of pile-driving sounds on juvenile salmon also indicate that the US interim criterion for SEL_cum_ (set at 187 dB re 1 µPa^2^s for fish > 2 g) may be relatively low. Field experiments with juvenile steelhead (*Oncorhynchus mykiss*) [43] and juvenile Coho salmon (*O. kisutch*) [44] did not show sound-induced injuries or mortality at SEL_cum_ exposures up to 194 dB (steelhead) [43] or 207 dB (Coho) [44]. A recent study examined barotrauma injuries in juvenile Chinook salmon (*O. tshawytscha*) in relation to SEL_cum_, SEL_ss_ and the number of pile-driving strikes, using the HICI-FT [23]. They developed a ‘Response Weighted Index’ (RWI) to quantify the number and severity of the injuries and recommended a RWI-value to use as biological criterion for juvenile Chinook. The corresponding acceptable exposure bounds include impulsive sounds ≤ 179 dB re 1µPa^2^s SEL_ss_ for 1,920 strikes and ≤ 181 dB re 1µPa^2^s SEL_ss_ for 960 strikes, combined with a SEL_cum_ ≤ 211 dB.

It is important to realise that the present study only focussed on lethal effects of sound exposure. The applied exposures may have caused damage to body tissues or hearing, which did not lead to death within the monitoring period, but could result in lower long-term survival. Sound exposure may also affect physiology or behaviour and hence predation and starvation risks. Besides further research on lethal effects in fish larvae of other species, we recommend future research on sub-lethal effects, varying from injuries to behavioural responses.

A statistically significant effect of sound exposure in experiments does not necessarily indicate a ‘biologically significant’ effect for the entire larval population. To assess the effect of pile-driving sound on the total larval population (in a certain area, at a certain time), dose-response relationships for specific sound metrics (e.g. SEL_cum_, L_z−p_) are required as these can be translated to distance from the sound source (using source models and sound propagation models). Furthermore, information on the spatial and temporal distribution of fish larvae in relation to water movements is required; this can be obtained by egg and larval transport modelling (e.g. [20]). We recommend closer examination of the role of different sound metrics and co-variables (e.g. depth), as this enables a better assessment of the impact at the population level.
